# Supplementary material for: Body fat prediction through feature extraction based on anthropometric and laboratory measurements
Source: PLoS One. 2022 Feb 22;17(2):e0263333. doi: 10.1371/journal.pone.0263333 (PMC8863283; doi:10.1371/journal.pone.0263333)
Supplement: Attachment — Submitted filename: Response to Reviewers Final.doc [file pone.0263333.s001.doc]

Dear Editors and Reviewers,

Thank you very much for reviewing and providing invaluable feedback on our manuscript titled “Body fat prediction through feature extraction based on anthropometric and laboratory measurements” (ID: PONE-D-21-28367). We have revised the manuscript based on the feedback provided. Your comments have helped further improve our paper, and they have also provided important guidance for future research in this domain. We sincerely hope that, after these changes, you will consider our paper worthy of publication.

Our responses to the reviewers’ comments along with the corresponding changes to the manuscript are given below.

Editor:

1. The "methods" section is unclear and detailed setup of each model used during experiments is not presented.

**Response:** Thank you. In Section 2.2, we have now provided diagrams for each method along with their explanation. We have also added a detailed discussion of the setup for each model in Section 3.2. To further improve the understanding of the procedure, we have provided a diagram of the experimental procedure based on grid search with cross validation in Fig. 8.

2. There is no deep analysis of feature extraction results.

**Response:** Thank you. We have now added some detailed analysis of feature extraction results in Section 3 of the revised manuscript. All changes made have been highlighted in red in the revised manuscript.

3. Presentation needs improvements: Fig. 7 and Fig. 9 are hard to read - please consider using log scale for vertical axis

**Response:** Thank you for the suggestions. We have updated Figs. 7 and 9 in the manuscript: they are larger and easier to read now.

4. Presentation needs improvements: Table 7 - please remove not needed zeroes from values

**Response:** We have removed unnecessary zeroes from the values as recommended.

5. Selection of simple models is not discussed and justified. Models performing dynamic (contextual) data processing could give better results even for simple NNs in comparison with solutions with static FA/PCA/ICA (please see e.g. Sigma-if neural network).

**Response:** Thank you. The main contribution of this study is that our results are new in the context of body fat prediction, and they could be the baseline for future studies. Results from improved models (e.g., using different data pre-processing methods or improving the model itself) could be compared with these baseline results in our future studies. This is our motivation and justification for selecting the simple models in our study. We have updated Section 1 with a discussion of our motivation. We have also added the reference for Sigma-if neural network in Section 4.

6. Conclusions are very shallow.

**Response:** Thanks. We have revised the Conclusion Section and we believe it is now more substantial than the previous version. All changes made have been highlighted in red in the revised manuscript.

Reviewer #1:

1. What's the motivation of this paper?

**Response:** Thank you. We have revised the Introduction section to emphasize the motivation of this paper. Details are also provided here:

Introduction

*The motivation of this study is to assess and compare different feature extraction methods for body fat prediction as well as provide a baseline for future research in related areas. It is worth pointing out that the results presented here are new in the context of body fat prediction. We also explore the optimal number of features used for each of the feature extraction methods while balancing accuracy and efficiency.*

2. Comment: Author didn't give detailed setup of each model.

**Response:** Thank you. We have added detailed setups for each model in Section 3.2. Details are also provided here:

*Section 3.2 Parameter settings*

*We used the grid search approach with cross-validation for parameter selection [63]. The settings used in our experiments, obtained after some tuning process, are listed in Table1.*

***Table 1.*** *Parameter settings for the prediction models, where #neurons is the number of neurons, #iterations is the maximum number of iterations, regularization is the regularisation parameter, σ^2^is the variance within the RBF kernel, #trees is the number of trees, and depth is the maximum depth of the tree.*

|  | *Grid search* | *Optimal parameters* |
| --- | --- | --- |
| *MLP* | *#neurons=[100, 500, 1000]*  *#iterations=[100, 500, 1000]* | *#neurons=500*  *#iterations=500* |
| *SVM* | *regularisation=[10, 100, 1000]*  *1/σ^2^=[0.001, 0.01, 0.1]* | *regularisation=10*  *1/σ^2^=0.001* |
| *RF* | *#trees=[10, 100, 1000]*  *depth=[3, 4, 5]* | *#trees=1000*  *depth=5* |
| *XGBoost* | *#trees=[10, 100, 1000]*  *depth=[3, 4, 5]* | *#trees=1000*  *depth=5* |

*A flowchart of different feature extraction methods used for body fat prediction based on K-fold cross validation with N repeated experiments is given in Fig. 8 to further clarify the procedure of our experiments. In the figure, K= 5 and N= 20; i.e., the experiments were repeated 20 times and each experiment was conducted based on5-fold cross validation.*

3. Comment: No deep analysis of feature extraction results.

**Response:** As recommended, we have provided an in-depth analysis of feature extraction results in Section 3 of the updated manuscript. Details are also provided here:

*Section 3.3.3*

*Table 3 presents the results obtained by the MLP, SVM, RF and XGBoost for body fat prediction with and without feature extraction. As shown in the table, the SVM, RF and XGBoost perform better than MLP. The performance of SVM and XGBoost is similar, whereas that of RF is the best in terms of accuracy. However, it is clear that, by incorporating feature extraction, the learning models can achieve higher prediction accuracy, stability and robustness in most cases. The XGBoost model with FA feature extraction generated the most precise and stable results, albeit taking longer computation time than the standalone XGBoost. Using the feature extraction method increases the computation time because feature extraction pre-processing also takes time, even though it is more efficient to train the prediction model with less input features. Among all the prediction models, XGBoost with FA for feature extraction shows the best prediction accuracy (MAE=3.433, SD=4.188 and RMSE=4.248), and the SVM with PCA obtained results in the shortest computation time (close to the standalone SVM).*

*Section 3.3.5*

*To investigate the impact of having a different number of anthropometric features on the prediction performance, we increased the number of extracted features from 6 (as calculated in Section 3.3.2) to 13 (the total number of input features) in this series of experiments. Tables 5, 6 and 7 show the results obtained by the MLP, SVM, RF and XGBoost using FA, PCA and ICA, respectively. As shown in Tables 5 - 7, in most cases, the accuracy (RMSE and MAE) and stability (SD and MAC) were not necessarily enhanced by extracting more features as the inputs of the learning models. Among the models being compared, XGBoost-FA performs the best for predicting the body fat percentage in terms of MAE, RMSE, SD and MAC, which means it is able to predict the body fat percentage with the highest accuracy and stability on the StatLib dataset.*

*It is critical to reduce the number of dimensions when the data size or the number of dimensions is large (big data scenarios). In addition, the prediction models with PCA outperform the corresponding versions with ICA in terms of all the metrics used. This might be due to the Gaussian distribution of the body fat dataset since PCA can process the Gaussian distribution data while ICA cannot. Fig. 10 depicts the comparative experimental results of the computation time for the MLP, SVM, RF and XGBoost using FA, PCA and ICA, respectively. The results show that XGBoost with FA is the fastest among the compared methods.*

*Fig. 10 also reveals that in some cases, the computation time increases with more features, which further highlights the importance of feature extraction in improving the efficiency. The computation time includes the time for feature extraction and 20 runs of five-fold cross validation, which means that when a different number of features are extracted, the time for feature extraction may also differ.*

*Section 3.4.3*

*Table 9 presents results obtained through the MLP, SVM, RF and XGBoost for bodyfat prediction with and without feature extraction. These results are consistent with those shown in Table 3, and show that ensemble models such as XGBoost performs better than the MLP and SVM. Similarly, results show that incorporating feature extraction into the prediction models enhances the body fat prediction accuracy. The XGBoost model with PCA feature extraction generated the most precise and stable results, as well as shorter computation time than the stand-alone XGBoost.*

*Section 3.4.5*

*The results with 38 features are used as the baseline. Analysing the results from Tables 11 - 13 reveals that the MLP, SVM, RF, and XGBoost with feature extraction performed similarly or better than their corresponding baselines in terms of all metrics with only half the features (19 features). This shows the potential of greatly improving the efficiency in real-world applications. In addition, analysis reveals that PCA is more suitable for extracting features for the body fat dataset than ICA. The reason could be that this body fat dataset has a Gaussian distribution and PCA is better suited for Gaussian-distribution data whereas ICA is better suited for non-Gaussian distribution data.*

4. Comment: section 2.2 is waste of paper. You can find all those in wikipedia or any ML tutorial books/blogs/etc.

**Response:** Thank you. We have attempted to simplify the discussion by removing some details in the updated manuscript. As this is an interdisciplinary journal, in addition to machine learning, the audience might also be from other fields such as health care. Therefore, in this paper, we have provided basic details to make it more accessible for those from a non-machine learning background. In addition, we want this paper to have some tutorial value, which is why we have included these details for the sake of completeness.

Reviewer #2:

1. Comment: The method section is poorly written and rather unclear on many points; You don't need to show every step/equation for well-known methods. Just summarize them and describe your settings (e.g., # of node, activation function, LR...)

**Response:** Thank you for the suggestion. In the revised manuscript, we have removed some unnecessary details and equations for well-known methods; instead, we have provided diagrams for each method to clearly explain them in Section 2.2. We have also added a discussion of the settings in Section 3.2. Details are also provided here:

*Section 2.2.1*

*The MLP is a type of ANN that generally has three different kinds of layers, including the input, hidden and output layers [47]. Each layer is connected to its adjacent layers. Similarly, each neuron in the hidden and output layers is connected to all the neurons in the previous layer with a weight vector. The values from the weighted sum of inputs and bias term are fed into a non-linear activation function as outputs for the next layer. Fig. 4 shows an example of MLP with three, two and one input, hidden and output neurons, respectively. We can see from the figure that the input layer has three input neurons (x_1_, x_2_, x_3_) and one bias term with a value ofb1. Their values, based on the inner product with the weight matrix, are fed into the hidden layer. In this step, the input is first transformed using a learned non-linear transformation – an activation function g(·) – that projects the input data into a new space where it becomes linearly separable. The outputs of two neurons in the hidden layer depend on the outputs of input neurons and a bias neuron in the same layer with a value ofb2. The output layer has one neuron that takes inputs from the hidden layer with the activation function, where f(****x****) is the feed-forward prediction value from an input vector* ***x****.*

*Section 2.2.2*

*We can see from Fig. 5 that, unlike the SVM for classification problems that classifies a sample into a binary class, the SVM regression fits the best line within a threshold value ε with tolerate errors (ξ_i_ and ξ^∗^_i_).*

*After solving the objective function in Eq. (2) using the Lagrangian function [53]and Karush–Kuhn–Tucker conditions [54], we …*

*Section 2.2.3*

*The RF, proposed by Ho [56], is a decision tree-based ensemble model. For body fat prediction, the RF regression model uses an ensemble learning method for regression. It creates many decision trees based on the training set [57]. By combining multiple decision trees into one model, the RF model improves the prediction accuracy and stability. It is also able to avoid overfitting by utilising resampling and feature selection techniques.*

*Section 2.2.4*

*The training procedure of XGBoost is depicted in Fig. 7. It can be seen from the figure that XGBoost is based on gradient boosting. More specifically, new models (decision trees) are built to predict the errors (residuals) of prior models (from f_1_ to the current model). Once all the models are obtained, they are integrated together to make the final prediction.*

*Section 3.2 Parameter settings*

*We used the grid search approach with cross-validation for parameter selection [63]. The settings used in our experiments, obtained after some tuning process, are listed in Table1.*

***Table 1.*** *Parameter settings for the prediction models, where #neurons is the number of neurons, #iterations is the maximum number of iterations, regularization is the regularisation parameter, σ^2^is the variance within the RBF kernel, #trees is the number of trees, and depth is the maximum depth of the tree.*

|  | *Grid search* | *Optimal parameters* |
| --- | --- | --- |
| *MLP* | *#neurons=[100, 500, 1000]*  *#iterations=[100, 500, 1000]* | *#neurons=500*  *#iterations=500* |
| *SVM* | *regularisation=[10, 100, 1000]*  *1/σ^2^=[0.001, 0.01, 0.1]* | *regularisation=10*  *1/σ^2^=0.001* |
| *RF* | *#trees=[10, 100, 1000]*  *depth=[3, 4, 5]* | *#trees=1000*  *depth=5* |
| *XGBoost* | *#trees=[10, 100, 1000]*  *depth=[3, 4, 5]* | *#trees=1000*  *depth=5* |

*A flowchart of different feature extraction methods used for body fat prediction based on K-fold cross validation with N repeated experiments is given in Fig. 8 to further clarify the procedure of our experiments. In the figure, K= 5 and N= 20; i.e., the experiments were repeated 20 times and each experiment was conducted based on5-fold cross validation.*

2. Comment: The task is a continuous prediction (body fat percentage), and how did you perform SVM/RF on continuous prediction tasks? Did you group the body fat percentage into different classes?

**Response:** Thank you for pointing this out. We have added a diagram for SVM regression and described the procedure in Section 2.2.2 of the revised manuscript. We have also included the description of RF as a regression model. Details are also provided here:

*Section 2.2.2*

*We can see from Fig. 5 that, unlike the SVM for classification problems that classifies a sample into a binary class, the SVM regression fits the best line within a threshold value ε with tolerate errors (ξ_i_ and ξ^∗^_i_).*

*After solving the objective function in Eq. (2) using the Lagrangian function [53]and Karush–Kuhn–Tucker conditions [54], we …*

*Section 2.2.3*

*The RF, proposed by Ho [56], is a decision tree-based ensemble model. For body fat prediction, the RF regression model uses an ensemble learning method for regression. It creates many decision trees based on the training set [57]. By combining multiple decision trees into one model, the RF model improves the prediction accuracy and stability. It is also able to avoid overfitting by utilising resampling and feature selection techniques.*

3. Comment: Can authors compare them with methods without feature extraction (just min-max normalization on raw features)? This is an important baseline.

**Response:** Thank you for this suggestion. We have added the baseline results (without using feature extraction) in the revised manuscript. In Case 1 (Tables 4-6), the results with 13 features are used as the baseline, whereas in Case 2 (Tables 7-9), the results with 38 features are used as the baseline. We have also added additional discussions in Sections 3.3.5 and 3.4.5. Details are also provided here:

*Section 3.3.5*

*To investigate the impact of having a different number of anthropometric features on the prediction performance, we increased the number of extracted features from 6 (as calculated in Section 3.3.2) to 13 (the total number of input features) in this series of experiments. Tables 5, 6 and 7 show the results obtained by the MLP, SVM, RF and XGBoost using FA, PCA and ICA, respectively. As shown in Tables 5 - 7, in most cases, the accuracy (RMSE and MAE) and stability (SD and MAC) were not necessarily enhanced by extracting more features as the inputs of the learning models. Among the models being compared, XGBoost-FA performs the best for predicting the body fat percentage in terms of MAE, RMSE, SD and MAC, which means it is able to predict the body fat percentage with the highest accuracy and stability on the StatLib dataset.*

*It is critical to reduce the number of dimensions when the data size or the number of dimensions is large (big data scenarios). In addition, the prediction models with PCA outperform the corresponding versions with ICA in terms of all the metrics used. This might be due to the Gaussian distribution of the body fat dataset since PCA can process the Gaussian distribution data while ICA cannot. Fig. 10 depicts the comparative experimental results of the computation time for the MLP, SVM, RF and XGBoost using FA, PCA and ICA, respectively. The results show that XGBoost with FA is the fastest among the compared methods.*

*Fig. 10 also reveals that in some cases, the computation time increases with more features, which further highlights the importance of feature extraction in improving the efficiency. The computation time includes the time for feature extraction and 20 runs of five-fold cross validation, which means that when a different number of features are extracted, the time for feature extraction may also differ.*

*Section 3.4.5*

*The results with 38 features are used as the baseline. Analysing the results from Tables 11 - 13 reveals that the MLP, SVM, RF, and XGBoost with feature extraction performed similarly or better than their corresponding baselines in terms of all metrics with only half the features (19 features). This shows the potential of greatly improving the efficiency in real-world applications. In addition, analysis reveals that PCA is more suitable for extracting features for the body fat dataset than ICA. The reason could be that this body fat dataset has a Gaussian distribution and PCA is better suited for Gaussian-distribution data whereas ICA is better suited for non-Gaussian distribution data..*

4. Comment: On page1 last row, what is "Lagrange multiplier measurements (features)"? Typo?

**Response:** It is indeed a typo. We have corrected this to "measurements (features)".

Reviewer #3:

1. State the novelty of the work.

**Response:** Thank you for your comment. We have updated Section 1 to discuss the novelty of our paper. Details are also provided here:

*Section 1 Introduction*

*The motivation of this study is to assess and compare different feature extraction methods for body fat prediction as well as provide a baseline for future research in related areas. It is worth pointing out that the results presented here are new in the context of body fat prediction. We also explore the optimal number of features used for each of the feature extraction methods while balancing accuracy and efficiency.*

2. Comment: Fix some notational inconsistencies, e.g., in section 2.2.1, stick with either e_i or error_i

**Response:** Thank you for pointing this out. We have revised all error_i to e_i in Section 2.2.1.

3. Comment: Explain why you think ICA, PCA, and FA are the most useful feature extraction one should use and why MLP, SVM, RF, and XGBoost are picked as the prediction methods.

**Response:** Thank you. We selected ICA, PCA, and FA because they the three most widely used feature extraction techniques and they are suitable for different data distributions. We applied MLP, SVM, RF, and XGBoost as the prediction methods because they have been successfully used in many real-world applications with promising performance. We have also updated the manuscript by including the discussions of feature extraction methods and prediction models in Sections 3.3.5 and 3.4.5. Details are also provided here:

*Section 3.3.5*

*To investigate the impact of having a different number of anthropometric features on the prediction performance, we increased the number of extracted features from 6 (as calculated in Section 3.3.2) to 13 (the total number of input features) in this series of experiments. Tables 5, 6 and 7 show the results obtained by the MLP, SVM, RF and XGBoost using FA, PCA and ICA, respectively. As shown in Tables 5 - 7, in most cases, the accuracy (RMSE and MAE) and stability (SD and MAC) were not necessarily enhanced by extracting more features as the inputs of the learning models. Among the models being compared, XGBoost-FA performs the best for predicting the body fat percentage in terms of MAE, RMSE, SD and MAC, which means it is able to predict the body fat percentage with the highest accuracy and stability on the StatLib dataset.*

*It is critical to reduce the number of dimensions when the data size or the number of dimensions is large (big data scenarios). In addition, the prediction models with PCA outperform the corresponding versions with ICA in terms of all the metrics used. This might be due to the Gaussian distribution of the body fat dataset since PCA can process the Gaussian distribution data while ICA cannot. Fig. 10 depicts the comparative experimental results of the computation time for the MLP, SVM, RF and XGBoost using FA, PCA and ICA, respectively. The results show that XGBoost with FA is the fastest among the compared methods.*

*Fig. 10 also reveals that in some cases, the computation time increases with more features, which further highlights the importance of feature extraction in improving the efficiency. The computation time includes the time for feature extraction and 20 runs of five-fold cross validation, which means that when a different number of features are extracted, the time for feature extraction may also differ.*

*Section 3.4.5*

*The results with 38 features are used as the baseline. Analysing the results from Tables 11 - 13 reveals that the MLP, SVM, RF, and XGBoost with feature extraction performed similarly or better than their corresponding baselines in terms of all metrics with only half the features (19 features). This shows the potential of greatly improving the efficiency in real-world applications. In addition, analysis reveals that PCA is more suitable for extracting features for the body fat dataset than ICA. The reason could be that this body fat dataset has a Gaussian distribution and PCA is better suited for Gaussian-distribution data whereas ICA is better suited for non-Gaussian distribution data.*

4. Comment: Why is this work confined only to a body fat prediction? Can it be used for any other prediction problem with only a large number of real-valued features?

**Response:** Yes, this work could potentially extend to other prediction problems with a large number of features, e.g., finance, engineering and healthcare. We have updated the Conclusion section to include this discussion. Details are also provided here:

*The accurate prediction of body fat is important for assessing obesity and its related diseases. However, researchers find it challenging to analyse the large volumes of medical data generated. The main purpose of this study is to analyse and compare the prediction effectiveness of four well-known machine learning models (MLP, SVM, RF and XGBoost) when combined with three widely used feature extraction approaches (FA, PCA and ICA) for body fat prediction. The results presented in this paper are new in the context of body fat prediction; they could, therefore, provide a baseline for future research in this domain.*

*The feature extraction results themselves are a novel contribution of this work. The results provided by XGBoost with PCA feature extraction could be used as the baseline for future research in related areas. In future studies, we will investigate ways to improve the feature extraction method specified for body fat datasets. Methods of improving the prediction model (e.g. an improved MLP [66]), using XGBoost with PCA as a baseline for body fat prediction, need to be investigated. It is also worth noting that the findings of this work could be applied to other prediction problems with a large number of features, e.g., finance, engineering and healthcare. Finally, we will explore other applications of analysing the body fat percentage. For example, applying domain knowledge to group body fat percentages into different disease classes in order to confirm the relationship between the body fat percentage and specific disease(s).*

Summing up, we have improved the manuscript to the best of our ability by taking into consideration the valuable comments and suggestions we received from the Editor and the reviewers. All our changes have been highlighted in red in the revised manuscript. We would like to thank the Editor and reviewers again for helping us improve our paper and hope the revised version is approved for publication.

We look forward to hearing from you.

Yours sincerely,

The Authors
